# The Dual Effect of Rho-Kinase Inhibition on Trabecular Meshwork Cells Cytoskeleton and Extracellular Matrix in an In Vitro Model of Glaucoma

**DOI:** 10.3390/jcm11041001

**Published:** 2022-02-15

**Authors:** Juliette Buffault, Françoise Brignole-Baudouin, Élodie Reboussin, Karima Kessal, Antoine Labbé, Stéphane Mélik Parsadaniantz, Christophe Baudouin

**Affiliations:** 1 Department of Ophthalmology III, Quinze-Vingts National Ophthalmology Hospital, IHU Foresight, 75012 Paris, France; alabbe@15-20.fr (A.L.); cbaudouin@15-20.fr (C.B.); 2 Institut de la Vision, Sorbonne Université, INSERM, CNRS, IHU Foresight, 75012 Paris, France; fbaudouin@15-20.fr (F.B.-B.); elodie.reboussin@inserm.fr (É.R.); karima.kessal@inserm.fr (K.K.); stephane.melik-parsadaniantz@inserm.fr (S.M.P.); 3 Department of Ophthalmology, Ambroise Paré Hospital, APHP, Université de Paris Saclay, 92100 Boulogne-Billancourt, France; 4 Department of Biology, CHNO des Quinze-Vingts, IHU Foresight, 75012 Paris, France

**Keywords:** glaucoma, trabecular meshwork, Matrigel, 3D culture, intraocular pressure, outflow, cytoskeleton, rho-kinase inhibitor, prostaglandin analog

## Abstract

The trabecular meshwork (TM) is the main site of drainage of the aqueous humor, and its dysfunction leads to intraocular pressure elevation, which is one of the main risk factors of glaucoma. We aimed to compare the effects on cytoskeleton organization and extracellular matrix (ECM) of latanoprost (LT) and a Rho-kinase inhibitor (ROCKi) on a transforming growth factor beta2 (TGF-β2)-induced glaucoma-like model developed from primary culture of human TM cells (pHTMC). The TGF-β2 stimulated pHTMC were grown and incubated with LT or a ROCKi (Y-27632) for 24 h. The expression of alpha-smooth muscle actin (αSMA) and fibronectin (FN), and phosphorylation of the myosin light chain (MLC-P) and Cofilin (Cofilin-P) were evaluated using immunofluorescence and Western blot. The architectural modifications were studied in a Matrigel^TM^ 3D culture. TGF-β2 increased the expression of αSMA and FN in pHTMC and modified the cytoskeleton with cross-linked actin network formation. LT did not alter the expression of αSMA but decreased FN deposition. The ROCKi decreased TGF-β2-induced αSMA and FN expression, as well as MLC-P and Cofilin-P, and stimulated the cells to recover a basal cytoskeletal arrangement. In the preliminary 3D study, pHTMC organized in a mesh conformation showed the widening of the TM under the effect of Y-27632. By simultaneously modifying the organization of the cytoskeleton and the ECM, with fibronectin deposition and overexpression, TGF-β2 reproduced the trabecular degeneration described in glaucoma. The ROCKi was able to reverse the TGF-β2-induced cytoskeletal and ECM rearrangements. LT loosened the extracellular matrix but had no action on the stress fibers.

## 1. Introduction

Primary open-angle glaucoma (POAG) is a leading cause of irreversible blindness. This optic neuropathy affected more than 50 million people worldwide in 2020 [1]. Its main risk factor is elevated intraocular pressure (IOP) [2]. The trabecular meshwork (TM), in the iridocorneal angle, is the main site of drainage of the aqueous humor, and its dysfunction results in IOP elevation. The TM is a complex, three-dimensional structure composed of multiple layers of extracellular matrix (ECM) covered with trabecular meshwork cells (TMC) [3].

In POAG, abnormal resistance is generated in the outflow pathway including the juxtacanalicular TM, the inner wall of Schlemm’s canal, and its basement membrane [4]. In the TM, increase in resistance is linked to a mixed mechanism, including loss of TMC and changes in their architecture and remodeling of the ECM [4,5,6,7]. Changes in the morphology and stiffness of juxtacanalicular TM cells have been described [6]. TGF-β2 is a profibrotic cytokine known to be involved in glaucoma pathophysiology. It is significantly elevated in the aqueous humor of patients with POAG [8]. TM exposure to TGFβ 2 has been used to induce ocular hypertension in animal models and cultured human anterior segment perfusion studies [9,10]. In vitro studies have shown that TGF-β induced the synthesis by TM cells of components of the ECM not degradable by metalloproteinases, which could lead to increased outflow resistance [11]. TGF-β2 also increases cell stiffness by the formation of cross-linked actin networks (CLANs) via the Rho-ROCK pathway [12].

However, this primum movens of the glaucomatous pathology is still rarely targeted by glaucoma treatments. The only demonstrated therapeutic strategy to stop the progression of the visual field deterioration in glaucoma is to reduce IOP [2,13]. Among the medical treatments, prostaglandin analogs (PGA) are the most effective. PGAs increase the aqueous humor outflow. The mechanism of the hypotonic action of PGA is still imperfectly understood. It is mainly due to a promotion of the aqueous humor outflow through the uveoscleral route. However, an action of PGA on the TM, acting by remodeling the ECM, was described [14,15]. In recent years, studies in the field aimed to develop medication that act directly on the trabecular cytoskeleton. Rho-kinase inhibitors (ROCKi) represent a new therapeutic strategy in glaucoma, which precisely target a major pathway involved in the modifications observed in the TM [16]. 

**Figure 1:**
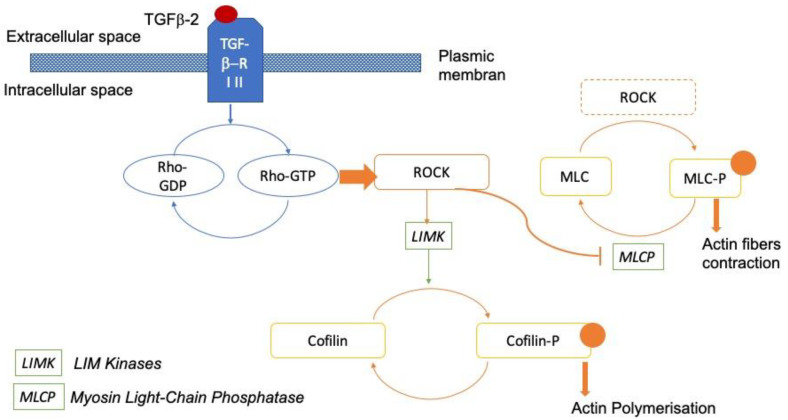
Rho-kinase signaling pathway. The TGF-β receptor (TRF-β RI-II) activates its effector molecules, ROCKs (Rho-kinases ROCK1 and 2). ROCKs inhibit myosin light chain phosphatase (MLCP). Phosphorylation of the myosin light chain induces actin fiber contraction. ROCKs activate LIM kinases, which phosphorylate cofilin, leading to actin stabilization [27].

The Rho GTPase/Rho kinase (ROCK) signaling pathway plays an important role in the modulation of the cytoskeleton of cells and the synthesis of ECM [16,17]. Rho GTPase activates its effector molecules, Rho-kinase ROCK1 and ROCK2. ROCK1 and 2 inhibit the myosin light chain phosphatase complex of Type 1 (MYPT1), thereby modifying the actin cytoskeleton. ROCK1 and 2 also activate LIM kinases (LIMKs), leading to the inhibition of Cofilin. This results in actin polymerization [18,19] (Figure 1). Activation of this pathway increases resistance to outflow, while its inhibition reduces IOP [12,20]. Rho-kinase inhibitors were recently approved for clinical use [21,22,23,24,25,26]. 

We aimed to study the effect of a ROCK-inhibitor on two major targets in glaucoma pathophysiology: the organization of both the trabecular ECM and TM cytoskeleton. We used a pathological TM model induced by TGF-β2 on 2D and 3D primary cultures of human TMC (pHTMC). Indeed, 2D cell culture models may not fully reflect the actual architecture of the TM. Cell culture in 3D could allow us to better mimic the microenvironmental conditions encountered in vivo. We compared the effects of ROCKi Y-27632 on the cytoskeleton of pHTMC and the TM ECM, with LT, the lead compound of the PGA family.

## 2. Materials and Methods

### 2.1. Primary Human Trabecular Meshwork Cell Isolation and Culture

Primary human TMC (pHTMC) was isolated from non-glaucomatous donor tissue rings. All donor tissues were obtained and managed following the guidelines of the Declaration of Helsinki for research involving human tissue. The human tissues used in our study came from corneoscleral rings discarded after the corneal graft. Human tissues were provided by the French eye bank, which ensured complete anonymization. The TM was carefully dissected under a microscope from a corneoscleral ring and transported in an Optisol-GS conservation medium (Bausch and Lomb Surgical, Inc.; Irvine, CA, USA) at room temperature (RT). The TM samples were digested with collagenase (Gibco^TM^ Collagenase, Type IV, Fisher Scientific, Cat. # 17-104-019) diluted to 10 mg/mL in culture medium for TMC (Trabecular Meshwork Cell Medium (TMCM), ScienCell Cat. # 6591, composition: basic medium, 2% fetal calf serum (FBS), 1% trabecular cell growth supplement, and 1% penicillin and streptomycin solution (penicillin 10,000 U/mL, streptomycin 10,000 μg/mL)) for 30 min at 37 °C and then stirred at RT for 30 min. After digestion, the cells were centrifuged at 1500× *g* for 5 min before being suspended in 250 μL of TMCM and seeded into a 24-well plate. Cells were seeded at 100,000 cells/mL (25,000 cells per well). The cultured cells were incubated at 37 °C in a humid atmosphere with 5% carbon dioxide. At 48 h, we added 250 µL of TMCM. Fresh culture medium was supplied every 3 to 4 days. Cells were maintained at 37 °C in a humidified atmosphere until 80–90% confluence was achieved, at which point cells were trypsinized using 0.05% Trypsin/0.5 mM EDTA (Gibco^TM^, Cat. #25300062, Grand Island, NY, USA) and subcultured at 1.8 × 10^5^ cells/mL in 25 cm^2^ or 75 cm^2^ cell culture flasks with TMCM. At each passage (P), a part of the pHTMC was seeded in 48-well plates for immunofluorescence analysis. Before use in experiments, all pHTMC strains were characterized by immunohistochemistry. All studies were conducted using cells before the 7th passage, and at least three different donors’ human primary cell cultures were used for each experiment. Information about pHTMC lines are available in supplementary data (Table S1).

### 2.2. Trabecular Meshwork Cell Characterization

Before use in experiments, all pHTMC strains were characterized by immunohistochemistry for expression of α-smooth muscle actin (alpha-SMA), aquaporin 1 (AQP1), chitinase-3-like 1 (CHI3L1), and CD44. Characterization was performed between the 1st and 4th passages. The induction of alpha-SMA in response to dexamethasone (DXM) at 100 nM for 7 days was also used. It represents a reliable marker for characterizing trabecular cells. The pHTMC were fixed with 4% paraformaldehyde for 15 min. Then, a saturation/permeation solution containing 0.1% Triton X-100 and 5% NGS in PBS was employed for 1 h at RT before incubation with specific primary antibodies overnight at 4 °C. Primary antibodies and dilutions used are listed in Table 1. After washing with PBS, the cells were incubated with the corresponding secondary antibodies (Thermo Fisher, donkey anti-mouse conjugated with Alexa fluor 594 (A21203), and donkey anti-rabbit conjugated to Alexa fluor 488 (A21206)), at a dilution of 1/1000 for 1 h at RT. The nuclei were stained with DAPI (1/1000 dilution).

Western immunoblot analysis of the myocilin induction in response to DXM was also used to characterize the pHTMC in accordance with the consensus recommendations [28]. The analysis of myocilin induction by DXM compared two conditions: 1/TMCM + 2.10^−3^% ethanol (control) for 6 days, and 2/TMCM + 100 nM DXM (Sigma D8893, stock solution 20 µg/mL) dissolved in ethanol for 6 days. Mouse anti-myocilin (Santa Cruz sc-137233, dilution 1/100), anti-β-actin (Cell signaling 3700, 1/5000) primary antibodies, and HRP-linked anti-mouse secondary antibody (Invitrogen) were used. Protein expression was analyzed using Quantity One software and normalized with β-actin protein.

### 2.3. Exposure to TGF-β2 and Therapeutic Molecules

pHTMC were seeded at 100,000 cells/mL in 48-well plates (100 µL/well; 10,000 cells/well); At subconfluence, TGF-β2 (5 ng/mL) was introduced in the TMCM and incubated for 24 h [29]. Then, the pHTMC were incubated for 24 h with TGF-β2 (5 ng/mL), combined either with the ROCKi Y-27632 (Santa Cruz Biotechnology) at 25 nM [30,31] or with Monoprost^®^ (LT) at 1/100 (i.e., 1.15 µM) (latanoprost 50 µg/mL, Laboratoires Théa, France). We used Y-27632 (25 nM), a ROCKi, which acts upstream of the phosphorylation of MYPT1 and Cofilin. The controls were vehicle (TMCM only) or TGF-β2 (5 ng/mL) for 48 h. 

### 2.4. Immunocytochemistry

The effects on the organization of the cytoskeleton and of the ECM were characterized in immunocytochemistry using the anti-alpha-SMA and the anti-fibronectin (FN) antibodies, respectively. Involvement of the Rho-kinase pathway was studied using the anti-Phospho-Myosin Light Chain 2 (Ser19) (MLC-P) and anti-Phospho-Cofilin (Ser3) (Cofilin-P). The pHTMC were fixed with 4% paraformaldehyde for 15 min. Primary antibodies and dilution used are listed in Table 2. After washing with PBS, the cells were incubated with the corresponding secondary antibodies (donkey anti-rabbit, conjugated to Alexa fluor 488 (Thermo Fisher, A21206)), at a dilution of 1/1000 for 1 h at RT. The nuclei were stained with DAPI (1/1000). Phalloidin (Alexa 546 (A22283) 1/200) was used to label actin filaments in the cytoplasm.

The immunofluorescence images were taken using a Nikon ECLIPSE Ti fluorescence inverted microscope; the images were acquired with 100× and 200× magnifications and then processed using ImageJ software (NIH, Bethesda, MD, USA).

The quantification was carried out using the Cellomics ArrayScanVTI (Thermo Fisher Scientific, MA, USA), an imaging system that detects, analyzes, and quantifies immunofluorescence staining on adherent cells. In each well, a central zone of 16 mm^2^ was analyzed. Using the HCS Studio Cellomics Scan software (Thermo Scientific, version 6.6.0), we measured the total area of positive labeling, which we related to the number of nuclei. An annular analysis pattern around each nucleus was drawn. The positive labeling area was measured in each of these rings. This area was then related to the number of nuclei.

### 2.5. Protein Extraction and Western Blot Analysis

The protein levels of fibronectin (FN) were quantified by Western blotting. Cellular proteins were extracted with ice-cold radioimmunoprecipitation assay (RIPA) buffer (RIPA Buffer, Sigma-Aldrich R0278) containing protease inhibitors (Complete Protease Inhibitor, Roche, Manheim, Germany) on ice. Proteins were quantified using a bicinchoninic acid assay (Thermo Fischer Scientific). Proteins from each sample (1 μg) were separated by electrophoresis on a NuPAGE™ 3–8% Tris-Acetate Protein Gel (Invitrogen EA03752PK2) in SDS running buffer (Invitrogen). The proteins were then transferred onto a polyvinylidene fluoride (PVDF) membrane and probed with the following primary antibodies: rabbit anti-fibronectin (Abcam ab2413, 1/500) and anti-β-actin (Cell signaling 3700, 1/1000). HRP-linked anti-rabbit secondary antibodies (Invitrogen) were used. Bound antibody was detected using Pierce™ ECL Plus Western Blotting Substrate (Thermo Scientific). Protein expression was analyzed by densitometry using ImageJ and normalized to the housekeeping proteins β-actin. 

### 2.6. Three-Dimensional (3D) Trabecular Meshwork Cell Culture 

For the 3D pHTMC culture, we used Matrigel^®^ (Corning Inc., Tewksbury, MA, USA), a basement membrane matrix secreted by Engelbreth–Holm–Swarm (EHS) mouse sarcoma cells [32]. Its composition is close to that of trabecular ECM, as it contains laminin-11, collagen IV, heparin sulfate proteoglycans, entactin/nidogen, and growth factors (FGF, EGF, TGF beta, IGF, and PDGF) [33]. The pHTMC stained with DiO (Vybrant™ DiO Cell-Labeling Solution, Invitrogen, V-22886) were gently mixed at a concentration of 10^5^ cells/mL in Matrigel^®^ diluted 1/2 in the TMCM culture medium and then sewn onto inserts in a 12-well plate (Greiner Bio-One ThinCert cell culture insert for 12 well plates, sterile, polyethylene terephthalate (PET) transparent membrane, pore diameter: 0.4 µm. Cat. N° 665641). The cells were incubated at 37 °C for 30 min, and then 800 μL of TMCM medium was added to the bottom of the well, and 200 μL in the inserts. The cells were then let incubate at 37 °C in a humid atmosphere with 5% CO_2_. Fresh culture medium was supplied every 3 to 4 days. The cells were exposed to TGF-β2 (5 ng/mL) for 48 h and LT and Y-27632 for 24 h, according to the same protocol as for the two-dimensional model. At 7 days, the 3D cultures obtained were fixed with 4% paraformaldehyde for one hour. Actin was stained with phalloidin (1/100), and the nuclei were stained with DAPI (1/1000) before analysis using confocal microscopy. Confocal laser scanning microscopy was performed using an Olympus IX81 confocal microscope coupled to Fluoview software (Olympus, Ver 4.2), and the images were acquired at 200× magnification. Confocal 3D images were processed using Imaris3D^®^ software (Bitplane AG, Zurich, Switzerland). All confocal images from the same experiment were captured using the same laser intensity and gain settings, so that the intensities of different samples could be compared.

### 2.7. Statistical Analysis

At least three different donors’ human primary cell cultures were used for each experiment. Data are expressed as mean ± standard deviation. The differences between vehicle-treated (controls), TGF-β2-treated, and TGF-β2/LT- or TGF-β2/Y27632-treated pHTMC were analyzed using ANOVA followed by Tukey’s multiple comparisons test (GraphPad Prism 9, LLC). *p* values < 0.05 were considered significant.

## 3. Results

### 3.1. Trabecular Meshwork Cell Characterization

Experiments conducted to validate our pHTMC culture are available in the supplementary data (Figure S1). There are no specific markers for trabecular cells, so we used a set of molecules known to be expressed by trabecular cells. pHTMC expressed alpha-SMA in relation to their important contractile property in the mechanotransduction process [3]. As expected, the AQP1 and CD44 antigens were located at the pHTMC plasma membrane. Nuclear localization of the protein CHI3L1 was found, compatible with the macrophagic activity of trabecular cells [34] (Figure S1b). Then, we used dexamethasone (DXM) exposure (100 nM) for 7 days to further characterize the pHTMC through alpha-SMA induction of and actin skeleton reorganization (Figure S1c). 

Western immunoblot analysis of the myocilin induction in response to DXM confirmed the TMC characterization in accordance with the consensus recommendations [28] (Figure S1d).

### 3.2. Exposure to TGF-β2

**Figure 2:**
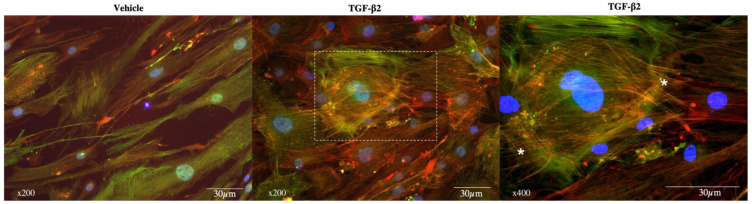
Cytoskeletal remodeling after TGF-β2 exposure. pHTMC were treated for 48 h with TMCM only (Vehicle) or with TGF-β2 (5 ng/mL). F-actin filaments were visualized by phalloidin staining (red) and alpha-SMA antibody (green), and nuclei were counterstained with DAPI (blue). Right: enlarged images from corresponding areas. Asterisks indicate CLANs.

**Figure 3:**
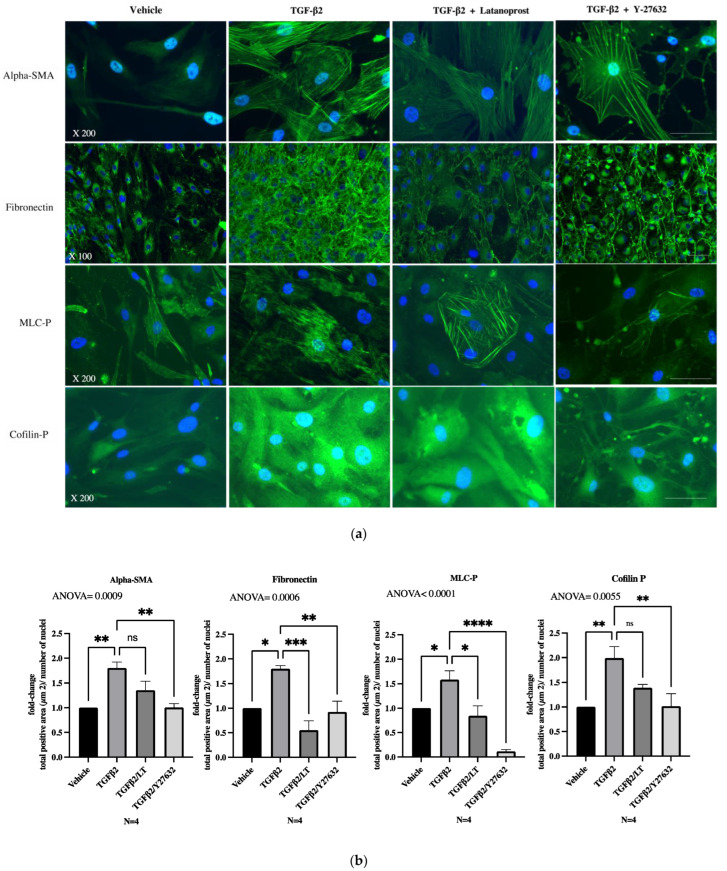
(**a**) Immunofluorescence analysis of the effect of LT 0.5 µg/mL or the ROCK-inhibitor Y-27632 25 nM for 24 h on pHTMC. pHTMC were treated for 48 h with TMCM only (Vehicle) or with TGF-β2 5 ng/mL or with 24 h of TGF-β2 5 ng/mL followed by 24 h of TGF-β2 5 ng/mL along with LT 0.5 µg/mL or Y-27632 25 nM. Nuclei are stained with DAPI (blue). The green staining corresponds to the antibodies indicated. Scale bar = 30 µm. This figure shows the major effect of LT on the density of ECM with no effect on the cytoskeleton itself. The ROCKi modified both the cytoskeleton and the ECM relaxation. (**b**) Quantification, with Arrayscan, of the total positive labeling area related to the number of cells (µm^2^) in fold-change (mean ± SEM). * *p* < 0.05, ** *p* < 0.01, *** *p* < 0.001, **** *p* < 0.0001.

Unlike the untreated primary trabecular cells, which displayed aligned actin fibers, after 48 h of exposure to TGF-β2 at 5 ng/mL, pHTMC exhibited rearrangements of the actin cytoskeleton, appearing disorganized and more extended. While labeling of alpha-SMA was more diffuse in the cytosol of untreated cells, there was a reorganization of fibers under the effect of TGF-β2. An increase in cell stress fibers was thus observed accompanied by the formation of CLANs (Figure 2 and Figure 3). These CLANs were present in 38.7% (±12%) of cells exposed to TGF-β2 and were not present in the control. Quantification of the alpha-SMA expression was also greater after treatment with TGF-β2 compared with the control (1.8-fold, *p* = 0.0116) (Figure 3b, alpha-SMA). 

The Rho-ROCK downstream signaling pathway for TGF-β2 induces phosphorylation of MYPT1, which inhibits the dephosphorylation of MLC and the phosphorylation of the intracellular protein Cofilin. After 48 h of exposure to TGF-β2 at 5 ng/mL, there was an activation of the ROCK pathway, with increased expressions of MLC-P and Cofilin-P compared to the vehicle-treated pHTMC (Figure 3b, MLC-P and Cofilin-P) (respectively 1.5 and 2.0-fold, *p* < 0.05). MLC-P immunofluorescence labeling presented the same distribution as actin: rather diffuse in the cytosol of untreated cells and organized into fibers after exposure to TGF-β2 (Figure 3a, MLC-P). Cofilin-P was located in the cytosol of pHTMC, and the staining was more intense after TGF-β2 exposure (Figure 3a, Cofilin-P).

Regarding the ECM, immunofluorescence analysis showed that following exposure to TGF-β2 at 5 ng/mL, fibronectin organized differently, with multiple joint fibrils forming a network that appeared thicker and denser than that found in the controls (Figure 3a, fibronectin). Quantification of the fibronectin expression was also greater after treatment with TGF-β2 vs. control (1.8-fold, *p* = 0.0119) (Figure 3b, fibronectin).

**Figure 4:**
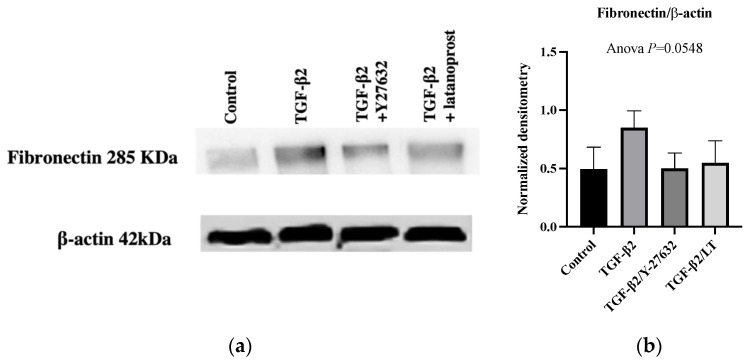
Protein expressions in pHTMC cultures after treatment with 5 ng/mL TGFβ2 for 48 h in the absence or presence of 25 nM Y27632 or 0.5 µg/mL LT for 24 h. (**a**) Representative Western blots of fibronectin and β-actin (mean ± SEM). (**b**) Densitometry of Western blot analysis of fibronectin normalized to β-actin.

Western blot analysis confirmed the induction of fibronectin expression in pHTMC cultures after TGFβ2 treatment compared with the control (Figure 4a,b).

### 3.3. Effects of Therapeutic Molecules on TGF-β2-Induced Pathological Trabecular Meshwork Model

Cytoskeletal rearrangements induced by TGF-β2 persisted under the effect of LT (Figure 3a, alpha-SMA). The expression of alpha-SMA remained more intense than in vehicle-treated pHTMC, which was confirmed after quantification using Arrayscan (Figure 3b, alpha-SMA) (ANOVA, *p* < 0.0001, TGFβ2 vs. TGFβ2/LT ns). CLANs were also present, and the expression profile of MLC-P and Cofilin-P did not differ qualitatively from TGFβ2-exposed cells in immunofluorescence images, even though the quantification showed a significant decrease in the total positive labeling area (Figure 3a,b). 

Regarding the ECM, following exposure to TGF-β2 at 5 ng/mL, fibronectin increased in density compared to that of the controls. LT greatly reduced the expression of fibronectin, resulting in the formation of a much looser mesh compared with TGF-β2-exposed pHTMCs (Figure 3a, fibronectin). The total positive area related to the number of nuclei was reduced (0.55-fold) when treating the TGF-β2-exposed pHTMC with LT (*p =* 0.0004) compared with TGF-β2-exposed pHTMC (Figure 3b, fibronectin). While non-significant, Western blot analysis revealed that the TGF-β2-exposed pHTMC with LT (0.5 µg/mL) seemed to decrease the TGF-β2-stimulated expression of fibronectin (ANOVA, *p* = 0.0548) (Figure 4a,b). 

The cytoskeletal rearrangements induced by TGF-β2 were modified by Y-27632. The intensity of α-SMA labeling was reduced compared to TGF-β2-exposed pHTMC (*p* = 0.017) (Figure 3b, alpha-SMA). Inhibition of ROCK was associated with relaxation of the cells and disassembly of stress fibers and CLANs. A new distribution of actin fibers in the periphery was observed in response to the alteration of the actin cytoskeleton (Figure 3a, alpha-SMA). TGF-β2-induced MLC-P and Cofilin-P overexpression were also inhibited by Y-27632 (*p* < 0.01) (Figure 3a,b, MLC-P and Cofilin-P). 

Regarding the ECM, the ROCK inhibitor Y-27632 decreased fibronectin expression, with a looser mesh and enlarged intercellular spaces compared with TGF-β2-exposed pHTMC (Figure 3a, fibronectin). The labeling quantification revealed a reduction in fibronectin expression compared to TGF-β2-exposed pHTMC (*p* = 0.0062) (Figure 3b, fibronectin). Western blot analysis showed that treating the TGF-β2-exposed pHTMC with the ROCK inhibitor Y-27632 (25 nM) for 24 h decreased the TGF-β2-induced overexpression of fibronectin (Figure 4a,b).

### 3.4. Three-Dimensional Trabecular Meshwork Cell Cultures

**Figure 5:**
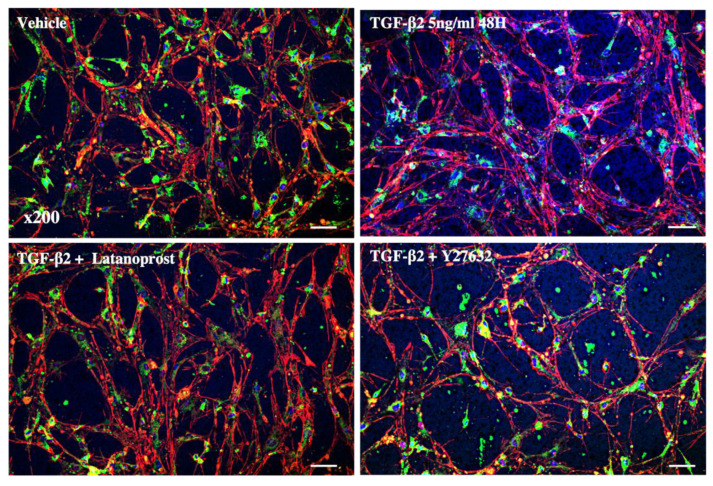
Confocal microscopy images of the 3D cultured pHTMC. Analysis of the effect of the ROCK inhibitor Y27632 at 25 nM for 24 h and the effect of LT at 0.5 μg/mL on primary human pHTMC treated with TGF-β2 at 5 ng/mL for 48 h. The cells were treated with TMCM alone (vehicle), 5 ng/mL of TGF-β2 for 48 h, or with TGF-β2 (5 ng/mL) for 24 h followed by a combination of TGF-β2 at 5 ng/mL and with Y-27632 (25 nM) or LT (0.5 µg/mL) for 24 h. Actin fibers are stained in red by phalloidin, membranes with DiO (green), and nuclei with DAPI (blue). Magnification 200×. Scale bar = 30 µm.

Figure 5 shows the 3D organization of pHTMC in Matrigel^TM^. The pHTMC organized in a mesh conformation with interconnections and the formation of intercellular spaces. Visual observations show that TGF-β2 induced rearrangements of the cytoskeleton with an organization of actin into more extensive fibers and decreased intercellular space. There was no modification of the actin disposition nor of the intercellular spaces after exposure to LT. However, the changes induced by TGF-β2 were modified under the effect of Y-27632. Actin fibers were less extensive, resulting in the widening of spaces between cells (Figure 5).

## 4. Discussion

In the present study, we used an in vitro TGF-β-induced pathological TM model from primary cultures of human trabecular cells. We first showed an effect of TGF-β2 on the organization of the cytoskeleton, with the formation of CLANs, and an increase in its contractibility, as well as an effect on the ECM, with an increase in fibronectin deposits. We also demonstrated activation of the ROCK pathway. We then showed that ROCKi has a dual effect on pHTMC with action on both the fibronectin deposition and the cytoskeleton, whereas latanoprost only acts on ECM degradation.

The family of TGF-β cytokines is known to be associated with impairment of several cellular functions, including differentiation, proliferation, and remodeling of the ECM [35]. The profibrotic role of TGF-β2 and its presence in the aqueous humor of patients with glaucoma implicates its potential role in the pathogenesis of ocular hypertension through TM degeneration/dysfunction [36]. In our study, we showed that TGF-β2 activated the Rho-ROCK pathway in TMC and induced actin fiber rearrangement and co-localization of alpha-SMA to stress fibers. TGF-β2 also induced fibronectin deposition. By simultaneously modifying the organization of the cytoskeleton and the ECM, with fibronectin deposition and overexpression, TGF-β2 allows the trabecular degeneration described in glaucoma to develop. TGF-β2 was also used in a TM model by Torrejon et al., who described the production of ECM and increased resistance to the aqueous humor outflow in vitro [37]. Ota et al. also showed that TGF-β2 enhances transendothelial electrical resistance in a culture of HTM [38]. Glucocorticoids such as dexamethasone are often used to model trabecular degeneration because they modify the cytoskeleton of TMC and the ECM. However, this corresponds more to the modifications obtained in corticosteroid-induced iatrogenic glaucoma, which constitutes a subtype of open-angle glaucoma [39]. This constitutes an advantage of the TGF-β2-induced model of TM alteration compared to the use of glucocorticoids. One of the limitations of our study is that we did not study the effect of therapeutic molecules in the absence of TGF-β2. Torrejon et al. studied the effect of Y-27632 alone on fibronectin expression of TMC and found no difference with the control. However, they demonstrated that Y-27632 in combination with TGF-β2 substantially decreased the expression of fibronectin compared to samples treated with TGF-β2 alone. Compared to vehicle control, TGF beta2/Y-27632 combined treatment increased fibronectin, demonstrating that the ROCKi counteracts the otherwise fibrotic effect of TGF-β2, effectively lowering ECM accumulation [37].

We highlighted an action of latanoprost on the fibronectin deposition without action on the cytoskeleton. This is consistent with the literature. Kalouche et al. showed that latanoprost decreased the accumulation of collagen onto cultured human trabecular cells [40]. Bahler et al. studied the effect of latanoprost on histologic sections of the anterior segment of the eye and observed focal losses of ECM in the juxtacanalicular region of the TM [15].

Moreover, in our study, we enriched our result on a three-dimensional (3D) TM cellular model. Our preliminary results presented an interesting tool to advance research on this pathology by taking into account biomechanics, which is a key element in the pathophysiology of glaucoma [41]. Nevertheless, for the moment, the results provided are only qualitative, which constitutes a limitation of the study. Quantification work is in progress. The 3D cell cultures in Matrigel^TM^ allowed us to obtain a meshed organization of trabecular cells with interconnections and the formation of intercellular spaces, which we did not find in 2D, and which better reflects the real anatomy of the TM. We were able to show with this model that the ROCKi Y-27632 widened the meshes between the cells by modifying the cytoskeleton of pHTMC. Cell culture in 3D recreates the conditions of the microenvironment encountered in vivo and provides cells with an environment allowing them to interact with each other and with the ECM. It would also help to better understand not only the physiological function of the TM but also its behavior under conditions of stress or toxicity, as well as the effect of medications [42]. This 3D TM model was first used by Bouchemi et al. to study the effect of benzalkonium chloride (BAK), a preservative commonly used in eye drops [29]. They showed that BAK induced inflammatory chemokines and inhibited the activity of MMPs, which play a crucial role in ECM degradation and increased outflow facility. Further research will be necessary to better exploit all the information provided by this model. Indeed, the main biomechanical cues experienced by TMC are not investigated in this article. For example, TMC in vivo are subjected to a significant pressure change, shear stress, and mechanical stretch. Rigidity and outflow measurement systems using this model might also be implemented to improve the relevance of the model. Analysis and understanding of the pathophysiology of the TM are essential for understanding and treating glaucoma.

We also showed the involvement of the ROCK signaling pathway in the stiffening of the TM and that the inhibitor Y-27632 modified the TGF-β2-induced cytoskeletal rearrangements. Previous studies have demonstrated the changes in pHTMC cytoskeleton organization with a modification of cell shape and actomyosin organization [43,44,45]. A new distribution of actin fibers in the periphery was observed in response to the alteration of the actin cytoskeleton. This redistribution was first described by Murphy et al. and qualified as cortical actin arrays (CAA) [46]. In our study, there was also reversibility of ECM deposition induced by TGF-β2 after treatment with ROCKi. Indeed, the ROCKi Y-27632 decreased TGF-β2-induced fibronectin deposition. A recent study by Li et al. also demonstrated the antifibrotic activity of a rho-kinase inhibitor on an in vivo glucocorticoid-induced ocular hypertension model [39]. Our work confirmed the anti-fibrotic action of ROCK inhibitors to prevent cell contractility and accumulation of ECM, consistent with studies by Torrejon et al., Pattabiraman et al., and Ota et al. [12,37,38].

Although it is known that the mechanism of action of PGA relies on increased expression of MMP in the TM, few studies have investigated the remodeling of the ECM by MMPs under the effect of ROCKi [47]. Torrejon et al. showed that after 3 and 5 days, TMC exposed to MMP2 mRNA-level in TMC was enhanced after a 3-days co-treatment with Y27632 and TGF-β2 [37]. Watanabe et al. showed that the addition of a pan-ROCKi (ripasudil 10 nM) to TGF-β2 (5 ng/mL)-exposed TMC induced significant up-regulation of MMP2, MMP9, and MMP14 at Day 6 [48]. Further studies exploring MMP and TIMP expression in TMC after PGA and ROCKi exposure would be of interest.

A ROCK inhibitor, 0.02% netarsudil (Rhopressa^®^ (US)/Rhokiinsa^®^ (EU), Aerie Pharmaceuticals, Inc., NC) received Food and Drug Administration approval in December 2017 and marketing authorization to the European Medicines Agency in November 2019 for lowering IOP in patients with POAG [49]. Another molecule from the same family, ripasudil (Glanatec^®^, Kowa Pharmaceuticals, Japan) was approved by the Japanese health authorities in 2014 for the treatment of glaucoma, or ocular hypertension, as a second line after prostaglandin therapy [50]. In addition, the combination of netarsudil with latanoprost was clinically developed and led to a new formulation (Rocklatan^®^ (US)/Roclanda^®^ (EU), Aerie Pharmaceuticals, Inc., Bedminster, NC, USA), which had a greater effect on the reduction of IOP than either of its two components, by reducing IOP by an additional 1.8 mmHg on average compared to netarsudil, and 2.7 mmHg compared to latanoprost [51,52]. Although we have not tested the combination of the two molecules, our work suggests that the significant effect of latanoprost on fibronectin deposition associated with the remodeling of the ROCK inhibitor cytoskeleton could effectively lead to a significant decrease in resistance to the aqueous humor outflow.

## 5. Conclusions

We used an in vitro TGF-β2-induced TM remodeling mimicking glaucomatous trabeculopathy to confirm the effects of both a ROCKi and a PGA on the TM. We showed that ROCK inhibition had an action on the TM cells’ cytoskeleton by reducing actin stress fibers, as well as on ECM release. In our model, we also demonstrated that latanoprost loosened the ECM.

## Figures and Tables

**Table 1:** Primary antibodies used in immunofluorescence for the characterization of cells.

Antibody	Dilution	Host	Supplier	Reference
Alpha-SMA	1/100	Rabbit polyclonal	Abcam	ab 5694
CD44	1/125	Rabbit monoclonal	Abcam	ab 189524
Aquaporin 1 (AQP1)	1/100	Mouse monoclonal	Santa Cruz	sc 25287
Chitinase-3like 1 (CHI3L1)	1/125	Rabbit polyclonal	Thermo Fisher	PAS-43746

**Table 2:** Primary antibodies used in immunofluorescence staining.

Antibody	Dilution	Host	Supplier	Reference
Alpha-SMA	1/100	Rabbit	Abcam	ab5694
Fibronectin	1/100	Rabbit	Abcam	ab2413
Phospho-Myosin Light Chain 2 (Ser19)	1/100	Rabbit	Cell signaling	3671S
Phospho-Cofilin (Ser3)	1/100	Rabbit	Cell signaling	3313S

## Data Availability

The datasets used and/or analyzed during the current study are included in this published article or available from the corresponding author upon reasonable request.

## Supplementary Materials

The following supporting information can be downloaded at https://www.mdpi.com/article/10.3390/jcm11041001/s1. Table S1: HTMC lines information, Figure S1: Characterization of the normal human trabecular meshwork cells.
