# Supplementary material for: The Dual Effect of Rho-Kinase Inhibition on Trabecular Meshwork Cells Cytoskeleton and Extracellular Matrix in an In Vitro Model of Glaucoma
Source: J Clin Med. 2022 Feb 15;11(4):1001. doi: 10.3390/jcm11041001 (PMC8877133; doi:10.3390/jcm11041001)
Supplement: Supplementary file 1 [file jcm-11-01001-s001.zip › jcm-1555480-supplementary.pdf]

Supplementary Materials:

Table S1. HTMC lines information P: passage, IF: immunofluorescence, WB: Western blot.

HTMC line number	Age of the donor (years)	Characterization at passage	Used in experimental set (passage)
**1**	60	P1	IF (P3)
**2**	83	P1	IF (P3)
**3**	86	P1	IF (P4)<br>WB (P6)<br>Matrigel (P5)
**4**	62	P3	IF (P4, P6)
**5**	60	P3	WB (P6)
**6**	51	P2	IF (P3)<br>Matrigel (P5)
**7**	50	P2	IF (P3)
**8**	76	P2	IF (P6)<br>Matrigel (P6)
**9**	71	P2	IF (P6)<br>WB (P6)
**10**	49	P4	WB (P5)
**11**	74	P4	IF (P5)<br>WB (P5)<br>Matrigel (P6)

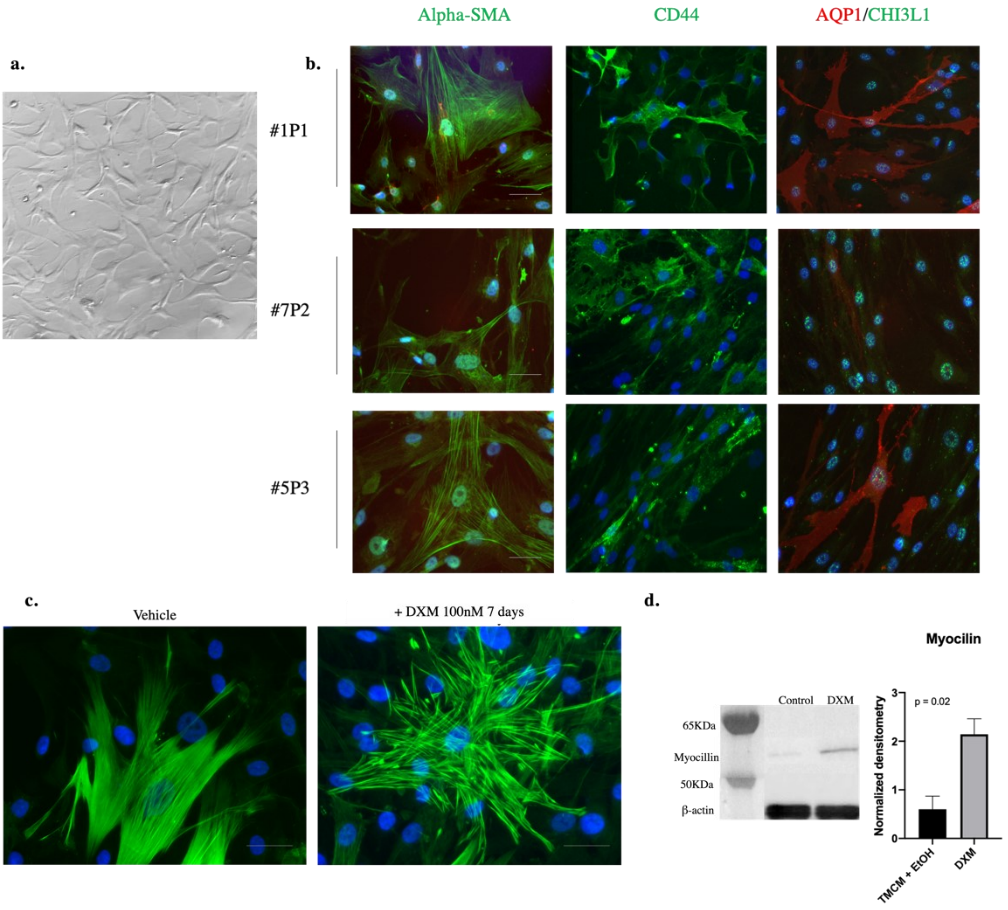

Figure S1. Characterization of the normal human trabecular meshwork cells a. Representative phase-contrast images of pHTMC on confluent phase (x20), TMC culture presented a cobblestone-like pattern with some overlapping processes. b. pHTMC from different donors (1 P1, 7 P2, 5 P3) were stained for markers expressed by TMC: alpha-SMA (green), CD44 (green), CHI3L1 (green), AQP1 (red) c. Immunolabeling of alpha-SMA (green), and nuclei (DAPI in blue) with or without exposure to dexamethasone (DXM, 100nM) for 7 days on pHTMC. The actin cytoskeleton is rearranged and there is an increase in the expression of alpha-SMA after exposure to dexamethasone. Magnification 200X, scale bar = 30 μm. d. Representative western blot of myocilin and β-actin and the densitometry analysis of myocilin normalized to β-actin showing dexamethasone (DXM)-induction of myocilin.
